# Treatment of extra-articular proximal and middle phalangeal fractures of the hand: a systematic review

**DOI:** 10.1007/s11751-017-0279-5

**Published:** 2017-03-04

**Authors:** D. Verver, L. Timmermans, R. A. Klaassen, C. H. van der Vlies, D. I. Vos, N. W. L. Schep

**Affiliations:** 1 Department of Surgery, Erasmus Medical Center, P.O. Box 2040, 3000 CA Rotterdam, The Netherlands; 2 Department of Surgery, Maasstad Hospital, P.O. Box 9100, 3007 AC Rotterdam, The Netherlands; 3 Department of Surgery, Amphia Hospital, P.O. Box 90157, 4800 RL Breda, The Netherlands

**Keywords:** Extra-articular phalangeal fractures, Fracture treatment, ORIF, Systematic review

## Abstract

The aim of the study was to systematically review the patient reported and functional outcomes of treatment for extra-articular proximal or middle phalangeal fractures of the hand in order to determine the best treatment options. The review methodology was registered with PROSPERO. A systematic literature search was conducted in electronic bibliographic databases. Two independent reviewers performed screening and data extraction. The evaluation of quality of the included studies was performed using the Structured Effectiveness Quality Evaluation scale. The initial search yielded 2354 studies. The full text manuscripts of 79 studies were evaluated of which 16 studies met the inclusion criteria. In total, 513 extra-articular proximal and middle phalangeal fractures of the hand were included of which 118 (23%) were treated non-operatively, 188 (37%) were treated by closed reduction internal fixation (CRIF) and 207 (40%) by open reduction internal fixation. It can be recommended that closed displaced extra-articular phalangeal fractures can be treated non-operatively, even fractures with an oblique or complex pattern, provided that closed reduction is possible and maintained. Conservative treatment is preferably performed with a cast/brace allowing free mobilization of the wrist. No definite conclusion could be drawn upon whether closed reduction with extra-articular K-wire pinning or transarticular pinning is superior; however, it might be suggested that extra-articular K-wire pinning is favoured. When open reduction is necessary for oblique or spiral extra-articular fractures, lag screw fixation is preferable to plate and screw fixation. But, similar recovery and functional results are achieved with transversally inserted K-wires compared to lag screw fixation.

Type of study/level of evidence: therapeutic III.

## Introduction

Phalangeal fractures account for approximately 18% of all upper-extremity fractures and are the most common fractures of the hand [1, 2]. The proximal phalanx of the long finger is fractured most frequently compared with the middle or distal phalanges [2, 3]. However, phalangeal fractures regularly result in unsatisfactory outcomes possibly because too often these phalangeal fractures are regarded as trivial injuries [4, 5].

Treatment of extra-articular middle and proximal phalangeal fractures of the hand is aimed at achieving solid bone union and restoring hand function. Various treatment methods including buddy strapping, splinting, closed reduction internal fixation (CRIF) with Kirschner-wires and open reduction internal fixation (ORIF) with plates and/or screws have been described. When selecting a treatment method, factors such as fracture classification, displacement, stability and whether it is an open or closed fracture have to be taken into account [6, 7].

However, there is no evidence-based consensus concerning the best treatment for extra-articular middle and proximal phalangeal fractures of the hand. This paper systematically reviewed the literature and assessed the patient reported and functional outcomes to determine the most favourable treatment options.

## Methods

This review was conducted and reported according to the Preferred Reporting Items for Systematic Reviews and Meta-Analyses protocols (PRISMA-P) [8]. A review protocol was drafted and registered on PROSPERO with number CRD42015026979. All of the following steps were performed by two independent reviewers (LT and DV). Disagreement was resolved by discussion.

### Eligibility criteria

Inclusion criteria were randomized controlled trials, case–control studies, cohort studies and case series (*n* ≥ 10) including adult and adolescent (≥14 years) patients treated for extra-articular proximal or middle phalangeal fractures of the hand and reporting patient reported and/or functional outcomes. English or German manuscripts were included exclusively. Studies describing distal phalangeal fractures, intra-articular fractures and/or pathological fractures were excluded in addition to reviews, animal studies, cadaver studies, case reports, surveys, editorials, commentaries, conference abstracts and letters.

Open fractures can be classified according to concurrent soft tissue injury [9]. Type I open fractures consist of a simple skin laceration, superficial skin injury and/or digital nerve injury, whereas type II open fractures consist of complete extensor tendon injury or extensive skin loss requiring reconstruction and type III consists of flexor tendon injury or combined extensor tendon injury and extensive skin loss requiring reconstruction. Studies including patients with closed or type I open fractures were included exclusively.

### Outcome measures

The primary outcome was validated patient reported outcome measures (PROMs) such as the Disabilities of the Arm, Shoulder and Hand (DASH) Score. Secondary outcomes included other patient reported outcomes such as satisfaction, pain and time to return to work and functional outcomes including total active range of motion (TAM), range of motion (ROM), grip strength, union, malunion, loss of reduction, secondary procedures, infection. A TAM of a typical finger is 260°, which is the sum of active flexion at the metacarpophalangeal (MCP) (normal range: 0°–85°), proximal interphalangeal (PIP) (normal range: 0°–110°) and distal interphalangeal (DIP) (normal range: 0°–65°) joints [10].

### Literature search and study selection

A search strategy was constructed with help of a clinical librarian by using descriptors that included synonyms for ‘phalanx fracture’, ‘proximal and/or middle phalanx’ and ‘fracture treatment’ in various combinations. Articles were sourced from Embase, Medline, Web-of-Science, Cinahl, Pubmed (the subset as applied by publisher, containing references not yet indexed by Medline), Cochrane, Lilacs, Scielo, Proquest and Google Scholar. The search was performed in August 2015. If the eligibility criteria were met, full manuscripts were procured and reviewed. Additionally, reference lists from included articles were examined for suitable studies.

### Data extraction

Data were extracted using a standardized data collection form that was developed according to the Cochrane guidelines [11]. Data collected included publication details (authors, year, journal), type of study (e.g. retrospective case series), demographic data (number of subjects, age, sex), follow-up period, the type of treatment applied and the described patient reported and functional outcomes. If necessary, the primary authors were contacted to retrieve further information. The level of evidence was determined using the Oxford Centre for Evidence-based Medicine Levels of Evidence (2011).

The evaluation of quality of the included studies was performed using the Structured Effectiveness Quality Evaluation scale (SEQES) [12]. The SEQES appraises the overall quality of a study based on study design, subjects, intervention, outcomes, analysis and recommendations. Each category has individual criteria that can be scored from 0 to 2. A score between 1–16 is regarded as low quality, 17–32 as moderate quality and 33–48 as high quality.

### Statistical analysis

A meta-analysis was planned but not performed due to heterogeneity between studies, varying methodology and lack of direct comparative results.

## Results

### Study selection

**Fig. 1:**
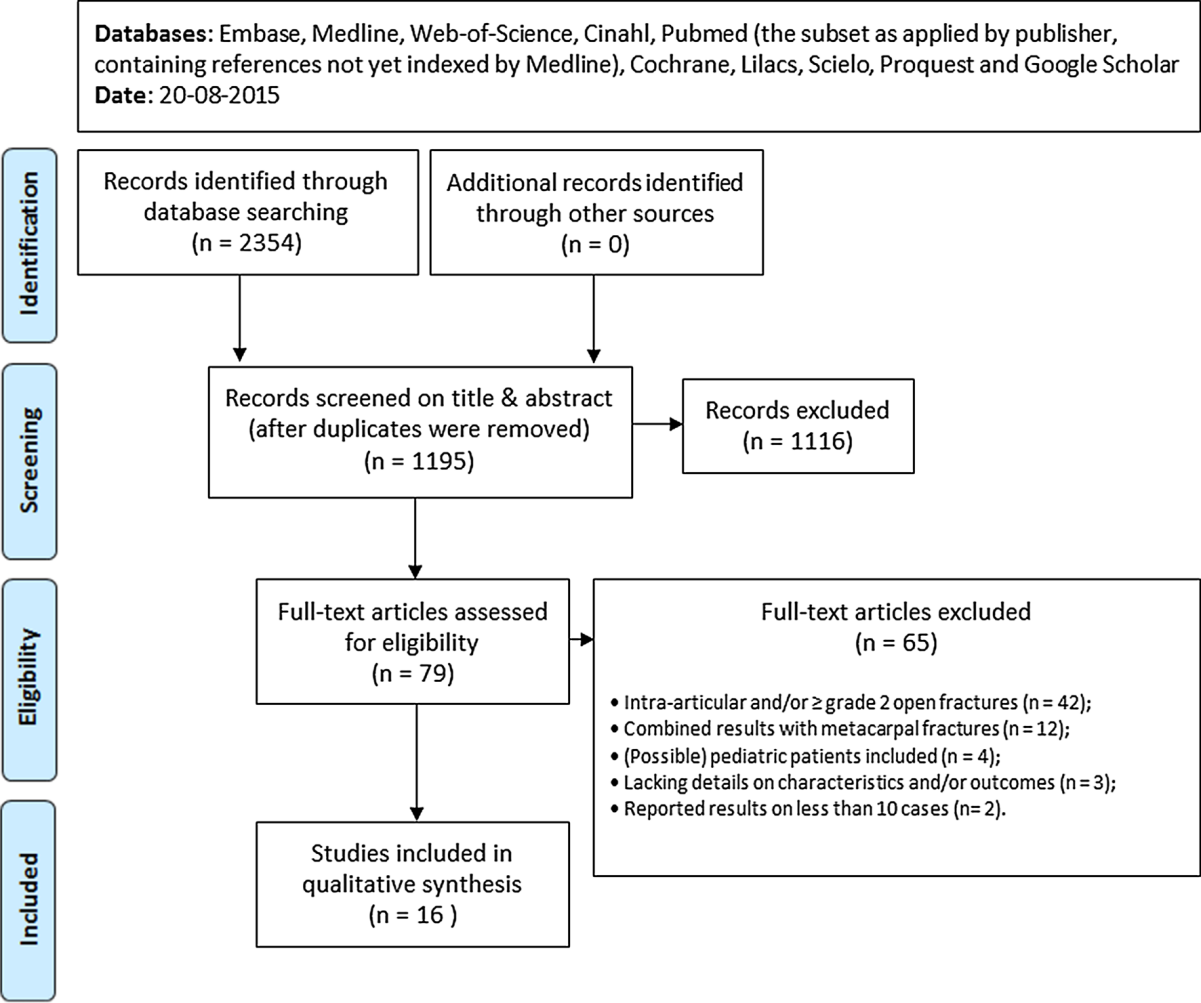
Flow chart

Figure 1 shows a flow chart depicting the study identification process. The initial search yielded a total of 2354 studies, of which 1195 remained after excluding the duplicates. The full text manuscripts of 79 studies were evaluated, and 16 studies were included in the systematic review. Examination of reference lists from included articles did not yield any additional suitable articles.

### Quality assessment

The SEQES scores varied from 15 to 31 with a mean of 21.7. Most studies (87.5%) were of intermediate quality. Table 1 illustrates a summary of SEQES scores of the included studies.

**Table 1:** Structured Effectiveness Quality Evaluation scale (SEQES)

References	Descriptors	Study design							Subjects				Intervention			Outcome			Analysis					Recommendations	Total^a^
	1	2	3	4	5	6	7	8	9	10	11	12	13	14	15	16	17	18	19	20	21	22	23	24	
Al-Qattan [23]	1	0	0	2	0	0	0	0	1	1	0	2	1	1	0	2	1	0	0	0	0	2	1	2	17
Al-Qattan [20]	1	2	0	0	0	1	1	0	2	2	0	2	1	1	2	2	1	0	2	1	1	2	1	1	26
Al-Qattan [24]	1	1	0	0	0	0	0	0	1	2	0	2	1	1	2	2	1	0	2	1	1	2	1	2	23
Al-Qattan [21]	1	2	0	1	0	1	1	0	0	2	0	2	1	1	2	2	1	1	2	0	1	2	1	1	25
Başar [13]	2	2	1	0	0	1	1	0	1	1	0	2	1	1	2	2	1	1	2	0	2	2	1	1	27
Brei-Thoma [25]	2	0	1	0	0	0	0	0	1	2	0	1	1	1	0	2	1	1	0	0	0	1	1	1	16
Faruqui [22]	1	2	0	0	0	1	1	0	1	2	0	2	1	0	2	2	1	1	2	1	0	2	1	2	25
Franz [17]	2	0	1	2	0	0	0	0	1	2	0	2	1	1	0	2	1	0	0	0	0	2	1	2	20
Franz [16]	2	2	1	2	1	1	1	0	1	2	0	2	1	1	2	2	1	0	2	1	1	2	1	2	31
Held [18]	2	0	1	1	0	0	0	0	1	2	0	2	1	1	0	1	1	0	0	0	0	2	1	1	17
Hornbach [14]	1	0	0	0	0	0	0	1	1	1	0	2	1	1	0	2	1	1	2	0	1	2	1	1	19
Horton [19]	1	2	1	2	2	1	1	1	2	2	0	1	1	1	2	2	1	1	2	0	1	1	1	2	31
Nalbantoǧlu [15]	1	0	1	0	0	0	0	0	1	1	0	2	1	1	0	2	1	1	0	0	0	2	1	2	17
Patankar [26]	1	0	1	2	0	0	0	0	1	2	0	2	1	1	0	2	1	1	0	0	0	2	1	2	20
Pehlivan [27]	1	0	0	0	0	0	0	0	1	1	0	2	1	1	0	2	1	1	0	0	0	2	1	1	15
Thomas [28]	1	0	1	0	0	0	0	0	1	2	0	2	1	1	0	2	1	1	0	0	0	2	1	2	18

^a^ Total points: 1–16 low quality, 17–32 moderate quality, 33–48 high quality

### Study and patient characteristics

Among the 16 included studies, there were two randomized controlled trials, four cohort studies and ten case series. In total, 513 extra-articular proximal and middle phalangeal fractures of the hand in 484 patients were included. The mean age of the included patients ranged from 22 to 49 years. Most patients were male (79%). A total of 118 fractures (23%) were treated non-operatively, 188 fractures (37%) were treated by CRIF and 207 fractures (40%) by ORIF. The mean follow-up time ranged from 7 weeks to 40 months. Details on the included fractures, the various applied treatment methods and the post-operative protocols are depicted in Table 2.

**Table 2:** Identified studies

References	Design	N^a^ (fract.)	P^b^	O^c^	Trauma	Fracture pattern	Type of treatment	Treatment details	LOE
Al-Qattan [23]	Pros. series	15 (15)	P1	0%	Industrial	Long oblique Spiral	Cerclage wires	Dorsal approach, longitudinally splitting extensor tendon, 2 or 3 pieces; light bandage overnight	IV
Al-Qattan [20]	Retro. cohort	78 (78)	P1	26%	Industrial	Transverse (100%)	K-wires (transarticular)	Antegrade or retrograde insertion (fixed MCPJ, free PIPJ), 2 pieces; palmar splint 10 days, K-wire removal 4–5 weeks	IV
							Interosseous loop wires	Dorsal approach/adjusting existing laceration, longitudinally splitting extensor tendon; light bandage overnight	
Al-Qattan [24]	Retro. series	35 (35)	P1	100%	Industrial	Transverse (100%)	K-wires (extra-articular)	Antegrade insertion (*N* = 20), mid-lateral approach with retrograde insertion (*N* = 15), 1 or 2 pieces; no splint, K-wire removal 5–6 weeks	IV
Al-Qattan [21]	Pros. cohort	20 (29)	P2	100%	Industrial	N.D.	K-wire	Insertion from tip of digit (fixed DIPJ, free PIPJ); K-wire removal 5 weeks	III
Başar [13]	Retro. cohort	22 (22)	P1	0%	N.D.	Oblique (64%) Spiral (36%)	Mini plate and screws	Dorsal approach, longitudinally splitting extensor tendon; splint 2 weeks	IV
							Screws only	Dorsal approach, longitudinally splitting extensor tendon, ≥2 pieces; splint 3 weeks	
Brei-Thoma [25]	Retro. series	25 (29)	P1	19%	N.D.	Basal transverse (50%) Transverse (28%) Oblique (17%) Spiral (6%)	Plate and screws, plate	Dorsal approach/adjusting existing laceration, longitudinally splitting extensor tendon; removable splint 4 weeks	IV
Faruqui [22]	Retro. cohort		P1	0%	N.D.	Transverse Short oblique	K-wires (transarticular)	Antegrade insertion (fixed MCPJ, free PIPJ), 2 pieces; splint 3–7 days, dorsal extension block splint 4 weeks, K-wire removal 3–4 weeks	IV
							K-wires (extra-articular)	Cross-pinning from radial and ulnar base, 2 pieces; splint 3–7 days, dorsal extension block splint 4 weeks, K-wire removal 3–4 weeks	
Franz [17]	Pros. series	15 (20)	P1	0%	N.D.	Transverse (45%) Oblique (30%) Spiral (25%)	LuCa	Metacarpal brace, dorsal extension PIPJs, free wrist, buddy tape	IV
Franz [16]	RCT	65 (74)	P1	0%	N.D.	Basal transverse (53%) Transverse (13%) Oblique (17%) Spiral (12%) Longitudinal (4%)	LuCa	Metacarpal brace, dorsal extension PIPJs, free wrist, buddy tape	II
							Forearm cast	Dorsopalmar plaster splint, dorsal extension PIPJs, fixed wrist, buddy tape	
Held [18]	Pros. series	23 (23)	P1	0%	Low velocity^d^	Transverse (39%) Oblique (39%) Complex (22%)	Dorsal splint	Dorsal plaster slab, buddy tape	IV
Hornbach [14]	Retro. series	11 (12)	P1	0%	N.D.	N.D.	K-wires (transarticular)	Antegrade insertion (fixed MCPJ, free PIPJ), 2 pieces; removable splint 3–4 weeks, K-wire removal 3–4 weeks	IV
Horton [19]	RCT	28 (28)	P1	N.D.	Sports, low velocity^d^	Long oblique Spiral	K-wires (transversal)	Transversal insertion, 2 or 3 pieces; palmar slab 3–4 weeks, K-wire removal 4 weeks	II
							Lag screws	Mid-lateral approach, excision lateral band extensor tendon, 2 pieces; splint 2 days, removable splint 3–4 weeks	
Nalbantoǧlu [15]	Retro. series	17 (18)	P1, P2	0%	Low- velocity^d^	Transverse (6%) Oblique (39%) Spiral (22%) Comminuted (33%)	Plate and screws and mini screws only	Dorsoulnar or radial approach w/o separating extensor tendon, ≥2 pieces; splint <3 weeks	IV
Patankar [26]	Pros. series	35 (35)	P1	0%	Industrial, sports, low velocity^d^	Transverse (40%) Short oblique (34%) Long oblique (20%) Segmental (6%)	Intra-medullary nailing	Dorsal approach, longitudinally splitting extensor tendon (*N* = 15), closed reduction (*N* = 20), 2 or 3 pieces; plaster slab 3 weeks	IV
Pehlivan [27]	Retro. series	23 (23)	P1, P2	0%	N.D.	Transverse (100%)	Tension band wiring	Dorsal approach, longitudinally splitting extensor tendon	IV
Thomas [28]	Retro. series	10 (10)	P1	100%	Road traffic accidents	Transverse (100%)	Theta fixation	Dorsal approach/adjusting existing laceration, longitudinally splitting extensor tendon; removable volar plaster 3 weeks	IV

*DIPJ* distal interphalangeal joint, *MCPJ* metacarpophalangeal joint, *N.D.* not described, *PIPJ* proximal interphalangeal joint

^a^Remaining patients and fractures in analysis after excluding patients lost to follow-up or with incomplete data

^b^Phalanx: proximal (P1) or middle (P2)

^c^Type I open fractures: without complete tendon injury or extensive soft tissue loss requiring reconstruction

^d^Low velocity: falling, straining, contusion

Only three studies of which one cohort study [13] and two case series [14, 15] evaluated validated PROMs. Başar [13] and Nalbantoǧlu [15] assessed disability of the hand/finger using the QuickDASH score, and Hornbach [14] evaluated general health using the Short-Form-36 (SF-36).

### Treatment

One RCT [16] and two case series [17, 18] comprised a total of 117 fractures in 103 patients treated non-operatively. One RCT [19], three cohort studies [20–22] and three case series comprised a total of 186 fractures in 176 patients treated with CRIF. One RCT [19], two cohorts [13, 20] and seven case series [15, 23–28] comprised a total of 198 fractures in 193 patients treated with ORIF. One RCT [19], one cohort study [20] and two case series [24, 26] reported on CRIF and ORIF. The outcomes per study are depicted in Table 3, and the pooled results of all studies are depicted in Table 4.

**Table 3:** Outcomes

References	Mean FU	Treatment	N	Outcomes								
				Mean TAM (range/SD)	TAM categories	NU	TAM <180°	TAM >240°	Inf.	Failure^a^	SSPs	Other/statistical outcomes
3A Non-operative treatment												
Franz [17]	12 weeks	LuCa	20	240° (155°–290°)	N.D.	0	0	12	N.A.	0	0	N.D.
Franz [16]	12 weeks	A: LuCa	44	246.6° (150°–300°)	N.D.	0	1	23	N.A.	2: loss of reduction	2: revision surgery (RS)	Mean TAM: NSD, *P* value N.D. Satisfaction (VAS): A 9.4 vs. B 8.4, *P* = 0.022* Mean wrist motion: A 128° vs. B 137°, *P* = 0.074
		B: Forearm cast	30	231.6° (145°–300°)	N.D.	0	1	14	N.A.	0	0	
Held [18]	7 weeks	Dorsal splint	23	N.D.	N.D.	0	N.D.	N.D.	N.A.	2: MU requiring RS	2: revision surgery (RS)	N.D.
3B Closed Reduction Internal Fixation (CRIF)												
Al-Qattan [21]	30 weeks	A: K-wire, w/o SSTC	16	241.3° (SD 8.5°)	Excellent (≥240°): 10 (63%) Good (210°–240°): 6 (38%) Fair (180°–209°): 0 Poor (<180°): 0	0	0	N.D.	0	0	0	Mean TAM: A vs. B, *P* < 0.001* Mean TTRBTW: A 15.1 weeks (SD ± 1.6) vs. B 26.8 weeks (SD ± 2.0), *P* < 0.001*
		B: K-wire with SSTC	13	186.9° (SD 20.7°)	Excellent (≥240°):0 Good (210°–240°): 3 (23%) Fair (180°–209°): 2 (23%) Poor (<180°): 7 (54%)	0	7	N.D.	0	0	0	
Faruqui [22]	8 months	A: K-wires (transarticular)	25	201°	N.D.	1	N.D.	N.D.	1	2: loss of reduction	5: tenolysis 1: capsulotomy	Mean TAM: NSD, P value N.D. Overall % complications (as defined by authors): A 56% vs. B 48%, NSD, P value N.D.
		B: K-wires (extra-articular)	25	198°	N.D.	0	N.D.	N.D.	0	0	1: tenolysis 1: capsulotomy	
Hornbach [14]	20 months	K-wires (transarticular)	12	265° (SD 25°)	N.D.	0	0	10	0	1: rotational MU requiring RS	1: tenolysis	SF-36: NSD compared to standardized values for general population Mean grip strength: compared to contralateral hand, P > 0.05
3C Open Reduction Internal Fixation (ORIF)												
Al-Qattan [23]	12 weeks	Cerclage wires	15	258° (245°–260°)	Excellent (> 75%): 17 (74%) Good (50–75%): 6 (26%) Fair (25–50%): 0 Poor (<25%): 0	0	0	N.D.	0	0	2: implant removal	N.D.
Başar [13]	19.2 months	A: Mini plate and screws	N.D.	212.3° (SD 30.3°)	N.D.	0	N.D.	N.D.	2	0	0	Mean TAM, A vs. B, *P* = 0.022* QuickDASH score: A 6.45 vs. B 2.58, *P* = 0.022* Loss of grip strength: A 6.1% (SD ± 8.6) vs. B 2.5% (SD ± 4.6), *P* = 0.1447 Mean TTRBTW: A 33.2 days vs. B 46.0 days, *P* < 0.05*
		B: Screws only	N.D.	235.0° (SD 25.6°)	N.D.	0	N.D.	N.D.	0	0	0	
Brei-Thoma [25]	10 months	Plate and screws, plate	29	213° (100°–285°)	Excellent (>250°): 7 (24%) Good (210°–250°): 11 (38%) Fair (180°–209°): 3 (10%) Poor (<180°): 8 (28%)	0	8	8	0	1: implant failure 2: rotational MU requiring RS	2: revision surgery (RS) 12: implant removal + tenolysis	N.D.
Nalbantoǧlu [15]	35 months	Plate and screws, mini screw only	18	200° (160°–260°, SD 39.5°)	Excellent (≥220°): 6 (33%) Good (180°219°): 5 (28%) Fair (130°179°): 7 (39%) Poor (<130°): 0	0	7	4	0	0	4: implant removal + tenolysis	Mean QuickDASH score: 3.4
Pehlivan [27]	13 months	Tension band wiring	23	N.D.	Excellent (> 75%): 17 (74%) Good (50–75%): 6 (26%) Fair (25–50%): 0 Poor (<25%): 0	0	0	N.D.	0	0	2: implant removal	N.D.
Thomas [28]	28.8 months	Theta fixation	10	N.D.	Excellent (> 250°): 9 (90%) Good (> 180°): 1 (10%) Fair (<180°): 0 Poor (no change): 0	0	N.D.	9^b^	0	0	3: implant removal	N.D.
3D CRIF and/versus ORIF												
Al-Qattan [20]	14 weeks	A: K-wires (transarticular)	40	N.D.	Excellent (> 240°): 5 (13%) Good (220–240°): 20 (50%) Fair (180–219°): 10 (25%) Poor (<180°): 5 (13%)	0	5	5	2	2: re-displacement	1: implant removal	TAM scores: A vs. B, *P* = 0.03* Mean TTRBTW: A 15 weeks vs. B 14 weeks, NSD, P value N.D.
		B: Interosseous loop wires	38	N.D.	Excellent (> 240°): 15 (39%) Good (220–240°): 16 (42%) Fair (180–219°): 3 (8%) Poor (<180°): 4 (11%)	0	4	15	0	3: re-displacement	0	
Al-Qattan [24]	16 weeks	C: K-wires (extra-articular)	35^c^	N.D.	Excellent (> 240°): 15 (43%) Good (220–240°): 10 (29%) Fair (180–219°): 5 (14%) Poor (<180°): 5 (14%)	0	5	15	4	0	0	Comparison with Al-Qattan 2008: TAM scores: A vs. C, *P* = 0.021* TAM scores: B vs. C, *P* = 0.599
Horton [19]	40 months	A: K-wires (transversal)	15	N.D.	N.D.	0	N.D.	N.D.	3	1: fixation failure	3: tenolysis 1: release of palmar plate	Mean (range) flexion PIPJ: A 81° (40–105) vs. B 80° (25–105), NSD, P value N.D. Mean (range) flexion DIPJ: A 55° (25–90) vs. B 49° (0–95), NSD, P value N.D. Median TTRBTW: A 3 weeks vs. B 1 week, NSD, P value N.D. Functional recovery: *P* = 0.3 Median VAS scores on pain and cold intolerance: NSD, P value N.D.
		B: Lag screws	13	N.D.	N.D.	0	N.D.	N.D.	3	1: fixation failure	0	
Patankar [26]	≥6 months	Intra-medullary nailing^c^	35^d^	N.D.	Excellent (≥85%): 35 (100%) Good (70–84%): 6 Fair (50–69%): 0 Poor (<50%): 0	0	0	N.D.	1	0	0	N.D.

*FU* follow-up, *Inf* infection, *MU* malunion, *N.A.* not applicable, *N.D.* not described, *NSD* not significantly different, *NU* non-union, *SSPs* secondary surgical procedures, *SSTC* significant soft tissue crush, *TTRBTW* time to return back to work

^a^As defined by authors

^b^TAM score of the remaining patient cannot be retrieved

^c^Of the 35 included patients, 15 were treated with open reduction and 20 patients with closed reduction, no differentiation made

**Table 4:** Pooled results

Treatment	*N*	Non-union	Poor TAM (<180°)	Good TAM (>240°)	Infection	Failure^a^	SSPs
Non-operative	117	0	2.1% (2/94)	52.1% (49/94)	N.A.	3.4% (4/117)	3.4% (4/117)
CRIF	186	0.5% (1/86)	14.8% (12/81)	28.8% (15/52)	4.1% (6/146)	3.2% (6/186)	7.5% (14/186)
ORIF	198	0	13.1% (19/145)	44.0% (51/116)	2.9% (5/175)	3.5% (7/198)	12.1% (24/198)
Total	501	0.2% (1/508)	10.3% (33/320)	43.7% (115/263)	3.4% (11/321)	3.4% (17/501)	8.4% (42/501)

*N.A.* not applicable, *SSPs* secondary surgical procedures

^a^Includes loss of reduction, fixation failures, unacceptable malunion (as defined by authors)

## Discussion

This systematic review provides an overview of the literature on treatment regimens used for closed or type I open extra-articular fractures of the proximal or middle phalanx of the hand in adolescent and adult patients, and outlines associated patient reported and functional outcomes. After a thorough search, available evidence was limited. Only two randomized controlled trials, four cohort studies and ten case series were included. The most important conclusions are depicted in Table 5.

**Table 5:** Most important conclusions

Conclusions	LOE
Non-operative treatment	
A cast/brace (with fixed MCP joints in 70°–90° flexion) allowing free mobilization of the wrist is preferred	II
Conservative treatment can also be used for closed displaced oblique or complex extra-articular fractures of the proximal phalanx, provided that closed reduction is possible and maintained, to achieve good functional results	IV
CRIF	
Patients with extra-articular fractures of the proximal phalanx in which initial non-operative treatment has failed can be successfully treated with CRIF	IV
ORIF	
Fixation with screw only, compared to plate and screws, is preferred in extra-articular spiral and oblique fractures of the proximal phalanx.	IV
CRIF vs. ORIF	
Similar recovery and functional results are achieved with transversally inserted K-wires compared to lag screw fixation in extra-articular fractures of the proximal phalanx	II

*LOE* level of evidence

### Outcomes non-operative treatment

There is no significant difference in radiological and functional outcome when a cast/brace (with fixed MCP joints in 70°–90° flexion) immobilizing the wrist is compared to a cast/brace without immobilization of the wrist (Level II, [16]). However, patients prefer a functional cast which enables free mobilization of the wrist as is shown by a significant higher score on a visual analogue scale (VAS) (Level II, [16]). Conventionally, almost all oblique, spiral or complex fractures were considered to be inherently unstable requiring internal fixation. However, it has been shown that closed displaced oblique and complex extra-articular fractures of the proximal phalanx from low-velocity injuries (including falling, straining, contusion) do not necessarily need to be treated with internal fixation, provided that closed reduction is possible and maintained, to achieve good functional results (Level IV, [18]).

### Outcomes CRIF

The degree of soft tissue crush is an important factor that influences both functional and patient reported outcomes as is shown by Al-Qattan [21]. He reported that industrial workers with extra-articular fractures of the middle phalanx with significant soft tissue crush have a lower active range of motion and take longer to return back to work (Level III). Faruqui [22] did not find a significant difference in active range of motion or complication rate (as defined by authors) between CRIF with transarticular (across the MCP joint) or extra-articular pinning used in closed extra-articular fractures of the proximal phalanx (Level IV). Patients with extra-articular fractures of the proximal phalanx in which initial conservative treatment has failed can be successfully treated with CRIF with transarticular inserted K-wires as is shown by Hornbach [14] (Level IV). This is noteworthy because the included articles on non-operative treatments all used (or anticipated to use) open surgery for correction in case of treatment failure.

### Outcomes ORIF

Başar [13] compared plate and screw versus screws only. They found a statistically significant difference in mean QuickDASH scores in favour of screw only fixation (2.58 versus 6.45); however, this difference (3.87) was lower than the established Minimal Important Difference (a score change that is related to a meaningful change in health status perceived by the patient) of 11 points [29]. Therefore, this difference may not be clinically relevant. Furthermore, finger range of motion was significantly more restricted in plate plus screw fixation in comparison with screw only fixation. On the other hand, patients with screw only fixation took significantly longer to return to work, but this was related to a longer period of immobilization that was necessary to prevent breaking of screws or loss of reduction. Nevertheless, it can be suggested that fixation with screw only is preferred in extra-articular spiral and oblique fractures of the proximal phalanx (Level IV).

### Outcomes CRIF versus ORIF

Open reduction with lag screws did not yield better functional results than closed reduction with transversally inserted K-wires in extra-articular fractures of the proximal phalanx. Also patients experience a similar functional recovery and time to return to work was comparable (Level II, [19]). Open reduction with interosseous loop wire fixation yields better TAM scores than closed reduction with transarticular K-wire fixation (across the MCP joint) in closed or type I open fractures of the proximal phalanx in industrial workers; however, time to return to work was similar (Level IV, [20]). Al-Qattan [24] found a better active range of motion in industrial workers treated with extra-articular K-wire pinning (with open or closed reduction) when compared to CRIF with transarticular K-wire pinning for type I open extra-articular fractures of the proximal phalanx (Level IV).

### CRIF with transarticular or extra-articular inserted K-wires

This systematic review showed contradictory results regarding CRIF using transarticular or extra-articular inserted K-wires. As stated earlier, Faruqui [22] did not find a significant difference in active range of motion or complication rate (as defined by authors) between CRIF with transarticular or extra-articular pinning used in closed extra-articular fractures of the proximal phalanx, whereas, on the other hand, Al-Qattan [24] did find a better active range of motion in industrial workers treated with extra-articular K-wire pinning (with open or closed reduction) when compared to CRIF with transarticular K-wire pinning. This might be explained by the fact that Al-Qattan [24] included patients with type I open fractures caused by industrial injuries. Those injuries are known to be associated with more crush and oedema. Hence, extra-articular K-wire insertion, which allows early mobilization of all joints, is expected to have better results in industrial injuries while the difference in closed extra-articular fractures caused by low-velocity injuries may not reach statistical significance. Both studies reporting on transarticular pinning have not assessed complications that might be induced by the potential cartilage damage that could be caused by transarticular pinning (across the MCP joint) such as secondary osteoarthritis or arthrosis. Thus, no definitive conclusions can be drawn upon whether transarticular pinning truly has similar complication rates as extra-articular pinning. Careful pinning is therefore always required when choosing transarticular K-wire fixation to minimize potential cartilage damage.

### Limitations

The first limitation of this review was the availability of only a few prospective comparable studies. The majority of the included studies were retrospective cohort studies and case series. The conclusions that can be drawn from these studies are limited by the lack of adequate control groups, and post-treatment complications are likely to be underestimated due to the retrospective design. In addition, the methodological quality of all the included studies was generally of moderate quality.

Second, many studies failed to provide specific information on the included patients and fractures (see Table 2 for detailed information regarding fracture patterns and involved phalanx). For example, most studies did not report on the type of injury that had caused the fractures and only one study specified the degree of soft tissue crush that was present. This lack of specifications may bias the results.

Thirdly, a large variation in reported outcomes was observed across the included studies. Some studies only reported on union, whereas others evaluated a mean TAM, extension lag in the PIP joint, grip strength, angulation in any plane, infection, satisfaction, time to return back to work, etc. This implies there is no general consensus which outcome measures (both patient reported and functional) we must focus on in order to conclude whether a certain treatment is effective and whether it is more preferable than another.

Also, the mean follow-up time between the studies included in this review varied considerable, namely from 7 weeks to 40 months. This adds difficulty in comparing treatments, especially when comparing outcomes such as treatment failure and secondary procedures because length of follow-up influences these results.

Another limitation that adds difficulty in comparing treatments is the lacking consensus on several definitions between studies. Eight studies reported on TAM scores that were categorized in groups ranging from excellent to poor. However, the definition per subgroup varied substantially. An excellent TAM score varied from >220° to >250° and a poor outcome varied from no change to <180°. Also, the definition of malunion and the degree of malunion that can be accepted before considering a corrective re-intervention varied across the included studies or was not defined at all. Some studies reported that any rotational malalignment was unacceptable, whereas others still accepted a rotational malalignment of 10°. Different degrees of angulation in any plane that were still accepted varied from 10° to 25°.

Last but not least, this review included both middle and proximal phalangeal fractures. However, most of the included studies were on extra-articular proximal phalangeal fractures. This must be taken into account when interpreting the results.

### Recommendations

The heterogeneity between the included studies made it impossible to adequately compare treatments and to demonstrate that one of the methods is superior. In future studies, there is need for consistency of definitions, treatment methods and structured follow-up for patients with extra-articular fractures of the proximal or middle phalanx of the hand. Despite the limitations of this systematic review, it can be recommended that closed displaced extra-articular proximal phalangeal fractures can be treated non-operatively, even fractures with an oblique or complex pattern, provided that closed reduction is possible and maintained. Conservative treatment is preferably performed with a cast/brace allowing free mobilization of the wrist. Although no definite conclusion could be drawn upon whether closed reduction with extra-articular K-wire pinning or transarticular K-wire pinning is superior, it might be suggested that extra-articular K-wire pinning is favoured. When open reduction is necessary, lag screw fixation is preferable to plate and screw fixation. But, similar recovery and functional results are achieved with transversally inserted K-wires compared to lag screw fixation.
